# Postmenopausal woman with 24 kilograms ovarian mucinous cystadenoma: a rare case report

**DOI:** 10.11604/pamj.2023.44.42.36942

**Published:** 2023-01-23

**Authors:** Dhruva Halani, Arpita Jaiswal

**Affiliations:** 1 Department of Obstetrics and Gynaecology, Datta Meghe Institute of Medical Sciences, Wardha, Maharashtra, India

**Keywords:** Ovarian cyst, mucinous tumour, postmenopausal, neoplasm, case report

## Abstract

A case of a 53-year-old postmenopausal woman presenting a giant ovarian cystic mucinous tumor weighing 24 kg is reported here. When she was seen first at our outpatient clinic, she had gross abdominal distension since 2 years, and she complained of unbearable aggressive pain. Her computed tomography (CT) scan was done which came suggestive of ovarian serous cystadenoma of large massive size 35 x 40 x 32 cm with moderate ascites. On exploratory laparotomy, a giant, totally cystic, vascularized and smooth mass attached to the right ovary was encountered. On the postoperative tenth day, she was discharged without any problem. Histopathology report of the right ovarian cystic mass came suggestive of multiloculated cyst with capsule intact with Borderline Mucinous tumor of right ovary weighing 24 kg. This is both one of the largest known examples in the literature and the largest ovarian cyst ever seen at our institution.

## Introduction

Rare ovarian tumors with dimensions larger than 10 cm are known as giant ovarian cysts (GOCs). Ovarian cysts are typically asymptomatic in the early stages and only manifest symptoms once they have grown to huge proportions, which makes early diagnosis difficult. Constipation, early satiety, vomiting, and frequent urination, growing abdominal distension, nonspecific diffuse abdominal pain, vaginal bleeding are symptoms of large ovarian cysts [1]. By origin cell type, ovarian neoplasms can be categorized into three primary categories: epithelial, stromal, and germ cell. Epithelial tumors are by far the most prevalent form when seen collectively. However, some research claim serous cystadenoma is the most prevalent benign ovarian tumor [2]. One of the frequent reasons of abdominal distention caused by the buildup of a lot of fluid is ascites, which is taken into account in the differential diagnosis. Cirrhosis of the liver and heart failure are frequent causes of transudative ascites. Less frequent causes include liver neoplasms, inferior vena cava or hepatic vein blockage, and constrictive pericarditis. When the ascetic fluid is exudative, the differential diagnosis includes pancreatitis, pelvic inflammatory illness, ruptured viscus, liver or peritoneal metastases, TB, and primary or secondary peritonitis caused by bile fluid. Hypothyroidism, endometriosis, collagen disorders, hypoalbuminemia, Meigs’ syndrome, pseudomyxoma peritonei, and leaking of the cysterna chyli or other lymphatic vessels are some more potential causes. Conditions that resemble ascites, such as diverticulum, hydronephrosis, pancreatic pseudocysts, and big tumors of the bladder, uterus and ovaries must also be considered [3]. We describe a patient with a rapid accumulation of fluid originally diagnosed as ascites by ultrasonographic examination, but which ultimately proved to be a borderline malignant giant ovarian cyst tumour

## Patient and observation

**Patient information and observation:** a 50-year-old female, P3L3, previous three normal delivery, postmenopausal since 2 years with known case of hypertension and hypothyroidism with sedentary lifestyle.

**Clinical findings:** the patient came with history of progressively growing abdominal edema, initially noted two years ago. Over six months, the abdomen had been swollen, which had been growing in size for the previous four months. She had anorexia and was unable to do basic household work because of the enormous mass. There was no prior history of gastrointestinal issues including colic, fainting spells, vomiting, or other gastrointestinal issues. Her bladder and bowel movements were regular. A history of widespread weakness existed.

**Figure 1:**
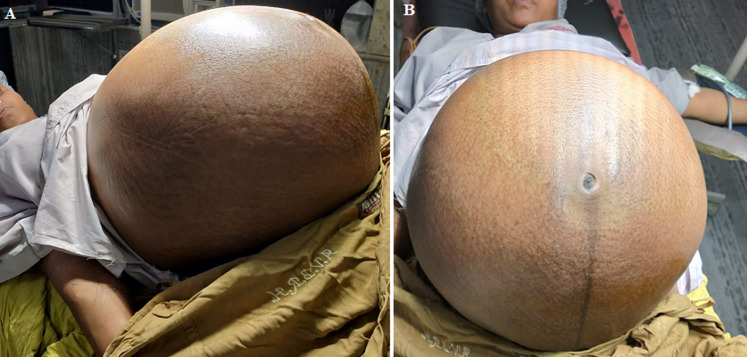
A,B) patient with massive ascites and large abdominal mass

**General examination:** she appeared undernourished and with a thin build. She was 60 kg in weight. She had bilateral pitting pedal edema, was normotensive, and had pallor. Upon abdominal examination, the abdomen was noticeably distended and with engorged veins. Forty-two inches of abdominal circumference measured at the umbilicus. In the flanks, bowel sounds could be detected. She was having 3 x 30 cm large ovarian cystic mass with uterus up to 34 weeks' size felt per abdomen (Figure 1A, B). Fluid thrill test was positive suggestive of the presence of ascites. A vaginal examination revealed cystocele, a right-sided deviated cervix flushed with vault, and fullness in both fornices.

**Diagnostic assessment:** on laboratory investigations, liver and kidney functions during preoperative testing were all normal. Her cancer antigen 125 (CA125) was 53.2 IU/ml and cancer antigen 19.9 was 1000 suggestive of ovarian tumor. Cardiomegaly and elevation of both diaphragm domes were visible on the chest X-ray. The uterus was bulky and was distinguished from the tumor on ultrasound. The entire abdomen appeared to be filled with a large cystic mass that had multiple septations. The mass forced pressure on internal organs of the abdomen. There was a dilation of the pelvicalceal system on the right side. Large (malignant) ovarian cysts and extensive ascites, tubo-ovarian mass, endometrial adenocarcinoma were identified as probable diagnosis. Her CT scan was done which came suggestive of ovarian serous cystadenoma of large massive size 35 x 40 x 32 cm with moderate ascites and proved confirmatory.

**Figure 2:**
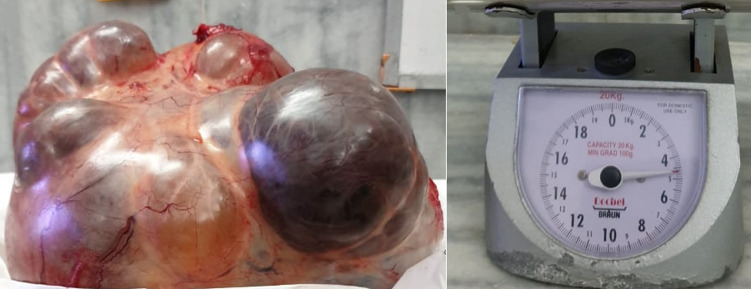
right ovarian cystic mass of 24 kgs

**Figure 3:**
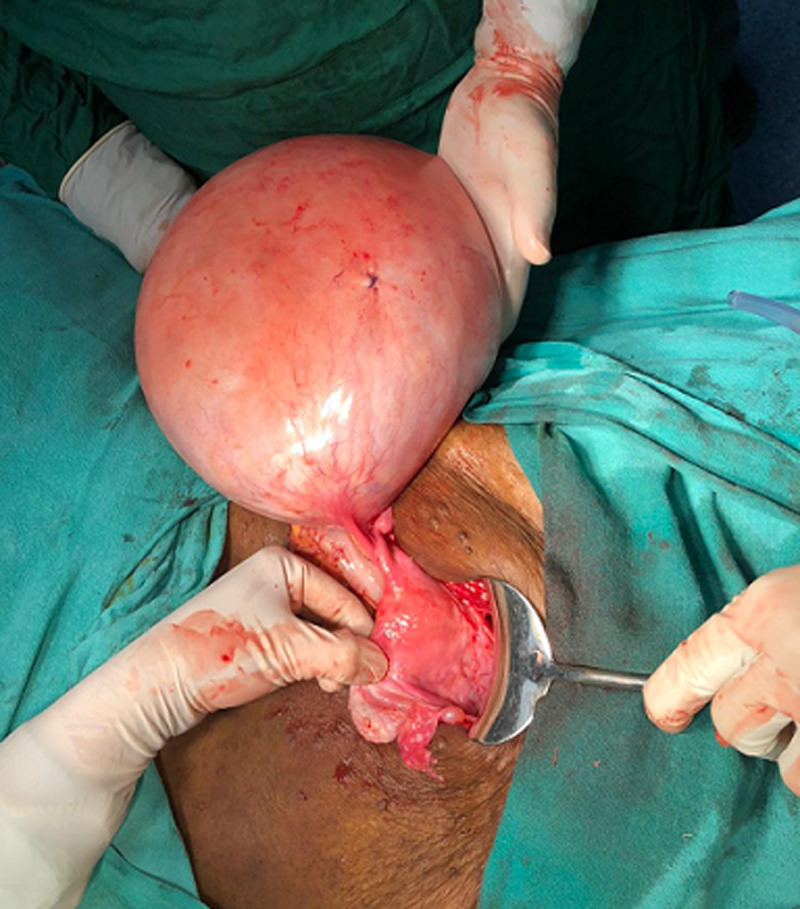
intraoperative 32 x 30 x 15 cm right ovarian cystic mass of 24 kgs

**Therapeutic intervention:** exploratory laparotomy was performed with simple total abdominal hysterectomy with bilateral salpingo-oophorectomy. An above-the-umbilicus vertical midline incision was used to access the abdomen. A tight smooth-surfaced cystic ovarian tumor spanning 30 x 32 x 16 cm that reached the diaphragm's underside was removed weighing 24 kgs with membranes intact (Figure 2, Figure 3). A suction of 18 liters of cystic fluid followed by simple hysterectomy was done. The right ovary is where the mass came from. The surface of the mass was regular, adhering to the right fallopian tube. The abdomen contained no free fluid. The mass was completely removed. Salpingo-oopherectomy and total abdominal hysterectomy were performed. Her postoperative care was done and the patient was vitally stable. Suture removal was done on day 10 postoperative. Histopathology report of the right ovarian cystic mass came suggestive of multiloculated cyst with capsule intact with borderline mucinous tumor of the right ovary.

**Follow-up:** the patient had no symptoms at all three months after the initial visit. Although she had put on weight, her physical examination revealed nothing abnormal.

**Patient´s perspective:** “I got relieved of the giant mass and the water in my abdomen and had the minimal hospital stay with less financial burden and did not have any cancer spread.”

**Informed consent:** informed consent was obtained from the patient

## Discussion

Nearly 40% of benign and 86% of malignant ovarian tumors are ovarian epithelial tumors, which account for about half of all ovarian malignancies. Cystic adenomas, adenofibromas, cyst adenofibromas, and surface papillomas are examples of benign serous tumors. These tumors are widespread, making up 58% of all ovarian serous tumors and around 25% of all benign ovarian neoplasms. Around 10% of all serous tumors are bilateral, 70% are benign, 5-10% are suspected to be borderline malignant, and 20-25% are malignant, mostly dependent on the patient's age [4]. Although unilocular serous cystadenomas are not infrequent, they typically have several lobes. They present as spherical or ovoid masses with a gross diameter of up to 35 cm [5], similar to the one described here. Due to financial limitations, our patient put off going to the hospital and decided against taking the chance of surgery; as a result, she came to us only after her condition had become completely incapacitating, and she was completely bedridden. According to Menahem *et al*. the case report mentioned a mucinous cyst borderline of nearly 22 kg [3]; Yeika EV *et al*. show giant ovarian cyst excision with membranes intact measuring 55 x 52 x 24 cm and weighing 10.8 kg [1]. Pre-operative or intraoperative drainage of giant ovarian cysts has also been proven unsafe and should be avoided. Einenkel J *et al*. [6] laid emphasis on that based on their critical evaluation of many medical cases and their complications. As imaging techniques advance and diagnoses are made earlier, ovarian tumors have grown rarer.

## Conclusion

Our patient presented late with such gross distention of abdomen only after morbid symptoms due to financial issues. Hence no early diagnosis could be made, which makes this case rare. This case report highlights the potential for large cystic ovarian tumors to present as significant massive ascites, necessitating the necessity to rule them out in all women presenting with ascites and to provide surgical intervention for its removal. An abdominal and pelvic ultrasound scan may help with the diagnosis in environments with limited resources.
